# Selective MIF Enolase Inhibitor TE-91 Regulates M1 Polarization and Associated Metabolic Reprogramming

**DOI:** 10.3390/antiox15050640

**Published:** 2026-05-18

**Authors:** Péter Deák, Nikoletta Kálmán, Csenge Antus, Eva M. Böhm, Marcell Krekó, Eszter Vámos, Viola Bagóné Vántus, Katalin Böddi, Lilla Makszin, Tamás Lóránd, Ferenc Gallyas, Balázs Radnai

**Affiliations:** 1 Department of Biochemistry and Medical Chemistry, University of Pécs, Medical School, H-7624 Pécs, Hungary; peter.deak@aok.pte.hu (P.D.); nikoletta.kalman@aok.pte.hu (N.K.); csenge.antus@aok.pte.hu (C.A.); eszter.vamos@aok.pte.hu (E.V.); viola.vantus@aok.pte.hu (V.B.V.); katalin.boddi@aok.pte.hu (K.B.); lorand.tamas@gmail.com (T.L.); ferenc.gallyas@aok.pte.hu (F.G.J.); 2 Otto-Loewi Research Center for Vascular Biology, Immunology and Inflammation, Division of Pharmacology, Medical University of Graz, A-8010 Graz, Austria; eva.boehm@medunigraz.at; 3 Department of Pharmaceutical Chemistry, Semmelweis University, H-1092 Budapest, Hungary; marcell.kreko@vichem.hu; 4 Institute of Bioanalysis, University of Pécs, Medical School, Szentágothai Research Center, H-7624 Pécs, Hungary; lilla.makszin@aok.pte.hu

**Keywords:** MIF, tautomerase, M1 polarization, oxidative burst, metabolic reprogramming, OXPHOS, glycolysis

## Abstract

Macrophage migration inhibitory factor (MIF) has been shown to induce M1 macrophage polarization with oxidative stress and associated metabolic reprogramming. Several tautomerase inhibitors were shown to selectively inhibit either MIF’s ketonase or enolase sub-activities. In this study, we aimed to investigate the role of enolase sub-activity in M1 polarization using the selective enolase inhibitor TE-91. We performed in silico molecular docking analysis and physicochemical characterization of TE-91. LPS + IFN-γ-induced RAW264.7 cells were applied as a model for M1 macrophage activation. We performed ROS and nitrite determinations, ELISA, qPCR, and immunoblot analysis, and measured mitochondrial oxygen consumption rate and extracellular acidification rate. Here, we reveal that TE-91 might directly bind to the MIF tautomerase active site. Furthermore, TE-91 reduces M1 activation by enhancing oxidative phosphorylation and reducing the glycolytic activity in LPS + IFN-γ-induced macrophage cells. In the same model, TE-91 reduces TNF-α, IL-6, CCL2, and iNOS mRNA transcription yet fails to modulate PARP1 and SOD2 mRNA transcription. It also decreases ROS, nitrite, and IL-6 production without influencing TNF-α and CCL2 protein production. TE-91 was unable to reduce either HIF-1α mRNA transcription or its protein expression. Finally, TE-91 reduced IL-1β cleavage, without affecting IL-1β protein expression. These results may highlight the importance of tautomerase sub-activities in M1 polarization.

## 1. Introduction

One of the main effectors and integrators of the immune system, macrophages fulfill a variety of functions. Among many additional functions they are phagocytes, transducers of biochemical signals and antigen-presenting cells [1]. Macrophages play a significant role during the initiation and progression of numerous inflammatory states, such as autoimmune myocarditis [2], ulcerative colitis [3] or atherosclerosis [4]. Thus, targeting macrophages is considered a promising therapeutic approach in the treatment of human inflammatory disorders. To achieve this goal, repolarization of macrophages became a new paradigm in anti-inflammatory therapies [5]. Macrophages can mainly be polarized into classically activated M1 cells and into alternatively activated, type II-activated or deactivated M2 cells. However, it must be stated that M1 and M2 macrophage polarization recently became considered oversimplified in macrophage activation [6,7]. In general, M1 macrophages exhibit proinflammatory characteristics, i.e., they induce Th1 responses, type I inflammation and produce reactive oxygen species (ROS) and proinflammatory cytokines such as tumor necrosis factor-α (TNF-α) or interleukin-6 (IL-6) for killing pathogens [6]. M1 polarization is accompanied by metabolic reprogramming of cells. It includes upregulation of aerobic glycolysis and the pentose phosphate pathway [8] together with a downregulation of mitochondrial oxidative phosphorylation [9].

Macrophage migration inhibitory factor (MIF) is one of the earliest discovered cytokines, which has been associated with inflammation, cancer and many additional human disorders. According to some recent studies, MIF is also involved in major depressive disorders [10], dry eye disease [11], intervertebral disk degeneration [12] and obesity [13]. MIF has several enzyme functions including keto-enol tautomerase activity. To determine the tautomerase kinetic parameters 4-hydroxyphenylpyruvate was primarily utilized as a model substrate [14]. However, MIF’s potential natural substrate in the human body is still unidentified. Despite the lack of this knowledge, tautomerase activity has a significant biological role. For example, proline 1 to glycine mutation (P1G) of the tautomerase active site of MIF was demonstrated to significantly decrease keto-enol tautomerase activity, thus reducing MIF’s cytokine functions [15]. Accordingly, MIF tautomerase was recently found to promote exosome-induced myeloid-derived suppressor cell (MDSC) formation in pancreatic cancer [16]. Moreover, contrary to P1G tautomerase-null mutant MIF, it increased gene expression supporting differentiation, activation and recruitment of MDSCs [16]. In another study, mice with MIF lacking tautomerase activity (MIF^P1G/P1G^ knock-in transgenic mice) were protected from high-fat diet-induced obesity. The genetic modification improved insulin resistance and reduced the inflammation in adipose tissue in mice [17]. Aligned with these findings, small molecule pharmacological MIF tautomerase inhibitors have been successfully evaluated and considered as potential anticancer and anti-inflammatory drugs [18].

In our previous studies, we presented the synthesis and evaluation of several MIF tautomerase inhibitors [19,20]. We determined the inhibitory effect of these compounds on each of the tautomerase sub-activities. Namely, on the ketone-to-enol conversion, which is referred to as ketonase activity and the enol-to-ketone conversion is termed as enolase activity. Additionally, we examined the anti-inflammatory properties of several selected compounds in lipopolysaccharide (LPS)-induced macrophage cells and in endotoxemic mice [19,20]. Astonishingly, we revealed compounds that were selective inhibitors of either the ketonase or the enolase sub-activities. For instance, we discovered the highly selective ketonase inhibitor KRP-6 significantly reduces MIF’s ketonase without affecting enolase activity. We found KRP-6 diminishes leukocyte migration, inflammatory M1 macrophage activation, and the associated metabolic reprogramming [21]. TE-11 is another potent non-selective MIF inhibitor, which reduces both ketonase and enolase sub-activities. It demonstrated similar effects in leukocytes and additionally improved Crohn’s disease-like colitis in mice [22].

In our recent study, we examined TE-91, a selective enolase inhibitor [20], and performed molecular docking analysis and physicochemical characterization of the compound. We also examined TE-91 on MIF-induced polymorphonuclear leukocyte (PMNL) migration and on M1 macrophage activation by using LPS + IFN-γ-induced RAW264.7 macrophage cells. In the latter model, we assessed mRNA transcription and protein expression of several inflammation-associated cytokines, enzymes and transcription factors. Again, in the same model, we analyzed M1 polarization-associated metabolic changes in macrophages by measuring oxygen consumption rate (OCR) and extracellular acidification rate (ECAR). The results were also compared with the effects of previously published MIF inhibitors, TE-11 and KRP-6.

## 2. Materials and Methods

### 2.1. Test Compound

The synthesis of the known compound ((*E*)-6-Hydroxy-2-(4-hydroxybenzylidene)-3,4-dihydronaphthalene-1(2*H*)-one [TE-91]) [23] was carried out by a three-step method utilizing the tetrahydropyran (THP) protective group to avoid potential oxidation of the starting hydroxyl-substituted aromatic aldehyde. THP comprises the protection of the aldehyde with dihydropyran. The product was then reacted in a base-catalyzed aldol condensation (under an inert atmosphere of argon) with 1-tetralone. Finally, during deprotection, the THP group was removed, yielding TE-91 [20], which was purified from methanol through recrystallization. The molecular structure, including the *E*-configuration of the arylidene side chain, was verified by spectroscopic methods. The physical data of our TE-91 sample used in the study were consistent in all respects with previously published results [20].

### 2.2. Molecular Modeling

The crystallographic structure of MIF in its complex with an inhibitor was obtained from the Protein Data Bank (PDB ID: 6B1K). All calculations were carried out with the modules originating from Schrödinger Suites 2019-2 (Schrödinger, LLC, New York, NY, USA) in Maestro. The protein was prepared by the addition of hydrogens and the missing side chains. Water, sulphate ions, and glycerol molecules, as well as ligands in the active sites formed by chains A-B and B-C, were removed. Hydrogen bonds were optimized at pH = 7.4, followed by minimization using the OPLS3e force field. The 3D structures of the ligands were determined by LigPrep at pH = 7.4 using the OPLS3e force field. The grid box for docking was centered on the ligand between chains A and C. Glide docking experiments were performed without any pharmacological constraints in XP mode. The polar surface area, octanol/water partition coefficient, and the Caco-2 and MDCK permeability values were predicted using QikProp as a module of Schrödinger 2019-2. Unionized molecules were utilized as inputs for the calculations.

### 2.3. Leukocyte Isolation

Phosphate-buffered saline (PBS, with 0.9 mM Ca^2+^ and 0.5 mM Mg^2+^, supplemented with 0.1% BSA, 10 mM HEPES, and 10 mM glucose; pH adjusted to 7.4) was prepared as an assay buffer. Peripheral blood mononuclear cell (PBMC) spin medium was acquired from pluriSelect Life Science (Leipzig, Germany), and the Eosinophil Isolation Kit from Miltenyi Biotec (Bergisch Gladbach, Germany). MIF was procured from PeproTech (London, UK), interleukin-8 (IL-8), and eotaxin/CC motif chemokine ligand 11 (CCL11) were from Immunotools (Friesoythe, Germany). Sterlitech (Auburn, AL, USA) provided PVP-free polycarbonate filters. All experiments utilizing human peripheral blood primary cells were approved by the Institutional Review Board of the Medical University of Graz (EK 17-291 ex 05/06). Human PMNLs were isolated from citrated whole blood donated from healthy volunteers. Erythrocytes were removed by dextran sedimentation, and PMNLs were separated from PBMCs by density gradient centrifugation utilizing PBMC spin medium. Eosinophils, up to a purity of ≥98%, were further separated from neutrophils of the PMNL fraction by negative magnetic selection via MACS Eosinophil Isolation Kit (Miltenyi Biotec).

### 2.4. Chemotaxis Assay

Human PMNL preparations were used to assess the migratory responsiveness of neutrophils [21]. Cells were resuspended in assay buffer, pre-treated with 20 µM KRP-6 or 20 µM TE-91 for 30 min at 37 °C and allowed to migrate towards MIF (3 nM) or IL-8 (10 nM) for another 60 min at 37 °C in a 48 well micro-Boyden chamber using PVP-free polycarbonate filters with a pore size of 3 µm. Migrated cells were enumerated by flow cytometry utilizing a BD Canto II flow cytometer (BD Biosciences, Franklin Lakes, NJ, USA). Acquisition was set for 30 s at a medium flow rate. Neutrophils were distinguished from eosinophils by their forward and side scatter properties and by autofluorescence.

### 2.5. Apoptosis Assay

Isolated PMNLs and purified eosinophils were pre-treated with 20 µM TE-91 for 60 min in RPMI 1640 (Fisher Scientific, Hampton, NH, USA) medium supplemented with 3% fetal bovine serum (FBS) and 1% penicillin-streptomycin (P/S). Following purification, 500 nM MIF or bovine serum albumin (BSA) in PBS (served as vehicle control) was added to the cells [21]. Following 24 h, cells were stained with allophycocyanin (APC)-annexin-V (1:100; BioLegend, San Diego, CA, USA) in the dark for 20 min at 4 °C and with propidium iodide (PI, 1:50; Sigma-Aldrich, St. Louis, MI, USA) in the dark for 1 min at room temperature. Samples were immediately analyzed via BD Canto II flow cytometer with the acquisition set for 60 s at medium flow rate. The total number of living cells (APC-annexin-V negative/PI negative), early apoptotic cells (APC-annexin-V positive/PI negative), and late apoptotic cells (APC-annexin-V positive/PI positive) were recorded.

### 2.6. Cell Culture and Treatments

We utilized the RAW264.7 mouse macrophage/monocyte cell line (ECACC, Salisbury, UK) during cell culture experiments. The cells were grown and cultured in Dulbecco’s Modified Eagle’s Medium (DMEM; Biosera, Cholet, France) containing 4.5 g/L glucose, *L*-glutamine, and sodium pyruvate (Biosera) supplemented with 10% endotoxin-free FBS (Corning Inc., Corning, NY, USA), without antibiotics. Cells were maintained up to 10 passages in 5% CO_2_ at 37 °C. On the day of experimentation, cells were plated onto TPP^®^ 96-well or 24-well plates (Sigma-Aldrich) or Seahorse XFp Cell Culture Miniplates (Agilent Technologies, Santa Clara, CA, USA) for overnight culturing. The following day, fresh medium was added, and cells were induced via 0.1 µg/mL LPS (*E. coli*, 01227:B8, Sigma-Aldrich) and 0.01 µg/mL interferon-γ (IFN-γ; Merck, Budapest, Hungary) or with 0.1 µg/mL LPS and 0.05 µg/mL TNF-α (PeproTech), respectively. LPS, IFN-γ, and TNF-α were diluted in the DMEM medium used. TE-91 was dissolved in dimethyl-sulfoxide (DMSO; VWR International, Debrecen, Hungary) and applied in 20 µM concentration as a 30 min pre-treatment before LPS + IFN-γ or LPS + TNF-α treatment. To rule out the effects of vehicle, control and treated groups received the same amount of DMSO in a dilution of 1:500.

### 2.7. Determination of Mitochondrial Bioenergetic Parameters

OCR and ECAR were detected via Seahorse XFp Extracellular Flux Analyzer (Agilent Technologies). First, RAW264.7 cells were plated onto Seahorse XFp Cell Culture Miniplates (Agilent Technologies) at a starting density of 2 × 10^4^ cells/well 24 h before the experiment. As described earlier, cells were pre-treated with 20 µM TE-91 for 30 min and then induced with 0.1 µg/mL LPS and 0.01 µg/mL IFN-γ for 8 h. Seahorse XFp Sensor Cartridges (Agilent Technologies) were hydrated overnight with XF Calibrant Solution (Agilent Technologies) and kept in a CO_2_-free incubator at 37 °C until the initiation of the measurements. Subsequently to pre-treatments, cell culturing media were replaced by Agilent XF Base (Agilent Technologies) unbuffered, serum-free assay medium (pH 7.4) supplemented with 10 mM glucose, 2 mM *L*-glutamine, and 1 mM sodium-pyruvate. To investigate mitochondrial functionality and bioenergetic parameters of cells, XFp Mito Stress Test kit (Agilent Technologies) was utilized, containing specific mitochondrial respiratory chain inhibitors, namely oligomycin, carbonyl cyanide-4(trifluoromethoxy)-phenylhydrazone (FCCP), and rotenone-antimycin A mixture in a final concentration of 1 µM, diluted in XF Base medium. ATP production rates from oxidative phosphorylation and glycolysis were calculated using Seahorse XFp Analyzer data and empirical conversion factors (Desousa et al., 2023) [24].

### 2.8. RNA Isolation and qPCR

RAW264.7 cells were seeded into a 24-well plate at a starting density of 5 × 10^5^ cells/well, pre-treated with 20 µM TE-91 for 30 min, and treated with 0.1 µg/mL LPS + 0.01 µg/mL IFN-γ for 24 h. Following cell collection, total RNA was extracted via MRX-03 MagCore^®^ triXact RNA kit 631 (RBC Bioscience Corp., New Taipei City, Taiwan) in full accordance with the manufacturer’s protocol. Extracted RNA was qualified using a Nanodrop 2000c spectrophotometer (Thermo Fischer Scientific, Waltham, MA, USA) and a Qubit 2.0 fluorometer (Thermo Fischer Scientific). A total of 2 µg of total RNA was reverse transcribed by Maxima First Strand cDNA Synthesis Kit (Thermo Fischer Scientific). A total of 100 ng cDNA was used in a final volume of 10 µL for real-time PCR with indicated primer pairs (Table 1) using Xceed qPCR SG 2x Mix (Institute of Applied Biotechnologies, Czech Republic) and CFX384 Touch Real-Time PCR Detection System (Bio-Rad, CA, USA). The ΔCt method was utilized for data analysis. RPL27 served as a reference for gene expression. Primers were acquired from Integrated DNA Technologies (Leuven, Belgium), in addition to TNF-α (Invitrogen, Carlsbad, CA, USA) (Table 1).

### 2.9. ROS Determination

To determine ROS production of macrophages, 10^5^ cells/well of RAW264.7 macrophages were seeded onto 96-well plates 24 h before the experiment. Macrophages received a pre-treatment with 20 µM TE-91 for 30 min and were subsequently induced with 0.1 µg/mL LPS and 0.01 µg/mL IFN-γ. At 24 h post-induction, dihydrorhodamine 123 (DHR123; Life Technologies, Carlsbad, CA, USA) was added in a 2 µM final concentration to the culturing media. After 1.5 h of incubation, the fluorescent intensity of the dye was measured via the Glomax Multi Detection System (Promega^®^, Madison, WI, USA).

### 2.10. Nitrite Measurement

To detect nitrite production of RAW264.7 macrophages, identical culturing and treatment conditions were utilized as for ROS determination. At 24 h post-induction, 50 µL of the supernatant was removed and mixed with an equal volume of Griess–Ilosvay reagent (Sigma-Aldrich) in a 96 well-plate. Optical density was determined by the Glomax Multi Detection System (Promega^®^) at a 550 nm wavelength.

### 2.11. Cytokine Production

In consideration of cytokine production, RAW264.7 cells were seeded at a starting density of 5 × 10^5^ cells/well and cultured in a 24-well plate. Following 24 h of incubation, macrophages received a 30 min pre-treatment of 20 µM TE-91 and then were induced with 0.1 µg/mL LPS and 0.01 µg/mL IFN-γ for TNF-α, IL-6, C-C motif chemokine ligand 2 (CCL2), and hypoxia inducible factor 1, alpha subunit (HIF-1α) measurements for an additional 24 h. Cytokine levels were determined from the culturing media via mouse TNF-α/IL-6/CCL2 uncoated ELISA kits (Invitrogen), or mouse HIF-1α pre-coated ELISA kit (FineTest Biotech Inc., Boulder, CO, USA), in full accordance with the manufacturer’s instructions. The absorbance was measured via the Glomax Multi Detection System (Promega^®^) at 450 nm. The cytokine concentration of the samples was calculated by using a calibration curve.

### 2.12. Immunoblot Analysis

For immunoblotting, RAW264.7 cells were pre-treated with either 20 µM of TE-91, TE-11, KRP-6, or DMSO for 30 min. Next, cells were induced for 24 h with 1 µg/mL mouse TNF-α along with 0.1 µg/mL LPS, in 6-well plates. Following washing with PBS, cells were collected in a RIPA lysis buffer (150 mM NaCl, 50 mM TRIS, 0.1% SDS, 1% Nonidet™ P40 Substitute, 0.5% Na-deoxycholate). Cell disruption was achieved through 10 s of sonication. Following centrifugation, the protein concentration of the lysates was measured via BCA Protein Assay Kit (Millipore, Burlington, MA, USA). Next, protein concentrations were normalized, and 5x Laemmli buffer was added to the samples. Lysates were then incubated at 98 °C for 10 min. A total of 13 μg of protein per sample was loaded onto the gels and blotted to nitrocellulose membranes at 250 constant mAs for 1 h at room temperature. Membranes were incubated overnight with anti-interleukin-1ß (IL-1ß) primary antibody (1:1000; Proteintech, Planegg-Martinsried, Germany), followed by incubation with Goat Anti-Rabbit IgG-HRP Conjugate (1:3000, BioRad, Hercules, CA, USA) secondary antibody for 80 min. Pierce™ ECL Western Blotting Substrate (Thermo Fisher Scientific) and Azure 300 imager (Azure Biosystems, Dublin, CA, USA) were utilized to develop and detect a chemiluminescent signal. The membranes were then stripped and reprobed with anti-GAPDH (TA308884, 1:20,000, OriGene, Rockville, ML, USA) primary and Goat Anti-Rabbit IgG-HRP Conjugate secondary antibody (1:3000, BioRad).

### 2.13. Trolox Equivalent Antioxidant Capacity Assay

During cell-free antioxidant capacity measurements, 2,2′-azino-bis(3-ethylbenzothiazoline-6-sulfonic acid) radical cation (ABTS^•+^) was used as a reducing agent, in which sample molecules were oxidized. The Trolox Equivalent Antioxidant Capacity of TE-91 was determined according to slightly modified standard protocols [25,26,27]. All assays were performed in a PBS solution. ABTS^•+^ radical cation was produced by reacting 2,2′-azino-bis(3-ethylbenzothiazoline-6-sulfonic acid) (Tokyo Chemical Industries, Tokyo, Japan) at 7 mM and potassium persulfate (Alfa Aesar, Haverhill, MA, USA) at a 2.45 mM concentration in water. The absorbance of the ABTS^•+^ solution was set between 0.7 and 0.75 at 754 nm using Ca^2+^ and Mg^2+^-free Dulbecco’s Phosphate-Buffered Saline (DPBS, Sigma-Aldrich). Trolox (Acros, Padborg, Denmark), as a reference substance, was dissolved in absolute ethanol, and TE-91 was dissolved in DMSO. Before spectrophotometric measurements, both Trolox and TE-91 samples were incubated with ABTS^•+^ solution at 37 °C for 6 min at a final concentration of 0, 0.3125, 0.625, 1.25, 2.50, 3.75, and 5 μM. DPBS served as a blank. The percentage inhibition of absorbance at 734 nm was calculated. All measurements were performed in triplicate for each concentration. Percentage inhibitions were plotted against the final concentration of the antioxidants. Slopes of TE-91 curves were compared with those of Trolox.

### 2.14. Radical Scavenging

Direct radical scavenging activity of TE-91 was measured as previously published [21]. Briefly, a cell-free system using the Fenton reaction was applied. A total of 100 µM H_2_O_2_ and 100 µM EDTA-Fe^2+^ salt were mixed with 2 µM DHR123 (Life Technologies) in PBS to induce the oxidation of the redox dye. TE-91 was diluted in PBS or DMSO and applied at a 20 µM final concentration to the system. Independently, DMSO was utilized in a 1:500 dilution (0.2%) as a vehicle control. The fluorescent intensity of DHR123 (494 nm excitation and 517 nm emission) was measured via the Glomax Multi Detection System (Promega^®^).

### 2.15. Macrophage Isolation

No in vivo animal experiments were performed in this study. We applied primary macrophage cells isolated from mice. To ensure full transparency, we have completed the relevant sections of the ARRIVE checklist. Mice were bred and maintained at the animal facility of the University of Pécs, Medical School, Department of Biochemistry and Medical Chemistry (permit number: ZOHU0104L-15). Macrophage isolations were performed in strict compliance with the European Communities Council Directive of 2010/63/EU. The animal facility was under the supervision of the Institutional Animal Use and Care Committee of the University of Pécs. For macrophage isolation, 16-week-old CD1 male mice (n = 6) weighing 35–40 g were anesthetized with 5% of isoflurane (Baxter Hungary Ltd., Budapest, Hungary) in 100% oxygen in an anesthetic chamber and sacrificed by cervical dislocation. For peritoneal macrophage (PM) isolation, ice-cold DPBS (Biosera) was injected into the abdominal cavity of the mice and removed with a 27 G needle. Next, cells were centrifuged, washed in DPBS, and resuspended in DMEM high glucose supplemented with 10% FBS and 1% P/S. Cells were plated onto a 96-well plate at a density of 10^5^ cells/well. The bone marrow-derived macrophage (BMDM) isolation was carried out according to Toda et al., 2020 [28]. Briefly, both the femur and tibia were removed from the mice’s lower limbs. The epiphyses were removed, and the bone marrow was washed out of the bones with ice-cold DPBS using a 27 G needle. Bone marrow was washed in DPBS, centrifuged, and resuspended in DMEM high glucose, supplemented by 10% FBS, 1% P/S, and 1 ng/mL macrophage colony-stimulating factor 1 (M-CSF1; Invitrogen). Cells were plated onto 96-well plates at a density of 2 × 10^5^ cells/well and differentiated into BMDMs for 7 days. One day before treatment, the media was aspirated, cells were washed with DPBS, and fresh media with no M-CSF1 was added. From here on, both PM and BMDM cell treatment and determination of ROS, nitrite, and inflammatory cytokines were carried out as aforementioned in Section 2.6, Section 2.7 and Section 2.8.

Raw data from the in vitro macrophage assays are available from the corresponding author upon reasonable request.

### 2.16. Statistical Analyses

All statistical analyses were performed via SPSS version 28.0 software (IBM, New York, NY, USA) or GraphPad Prism 10.0.3 software (Dotmatics, Boston, MA, USA). The normality of data distribution was investigated via Q-Q plot and/or box plot along with the Shapiro–Wilk test. A mixed-effects model, one-way ANOVA, Welch’s ANOVA, or two-way ANOVA with the appropriate post hoc tests were performed to compare the groups. The effect size of the ANOVA test was indicated as small, in which η2 was between 0.01 and 0.06, moderate, where η2 was between 0.06 and 0.14, and large, where the rank η2 was greater than 0.14. Student’s paired samples *t*-test was used to determine the statistical difference between the two groups. Effect sizes for the paired sample *t*-test were classed as very small where Cohen’s d value was less than 0.2, small where the d value was between 0.2 and 0.5, moderate where d was between 0.5 and 0.8, and large where d was greater than 0.8. *p*-values less than 0.05 were considered to be significant.

## 3. Results

### 3.1. TE-91 Binds to the Active Site of MIF Tautomerase by Two Orientations

**Figure 1:**
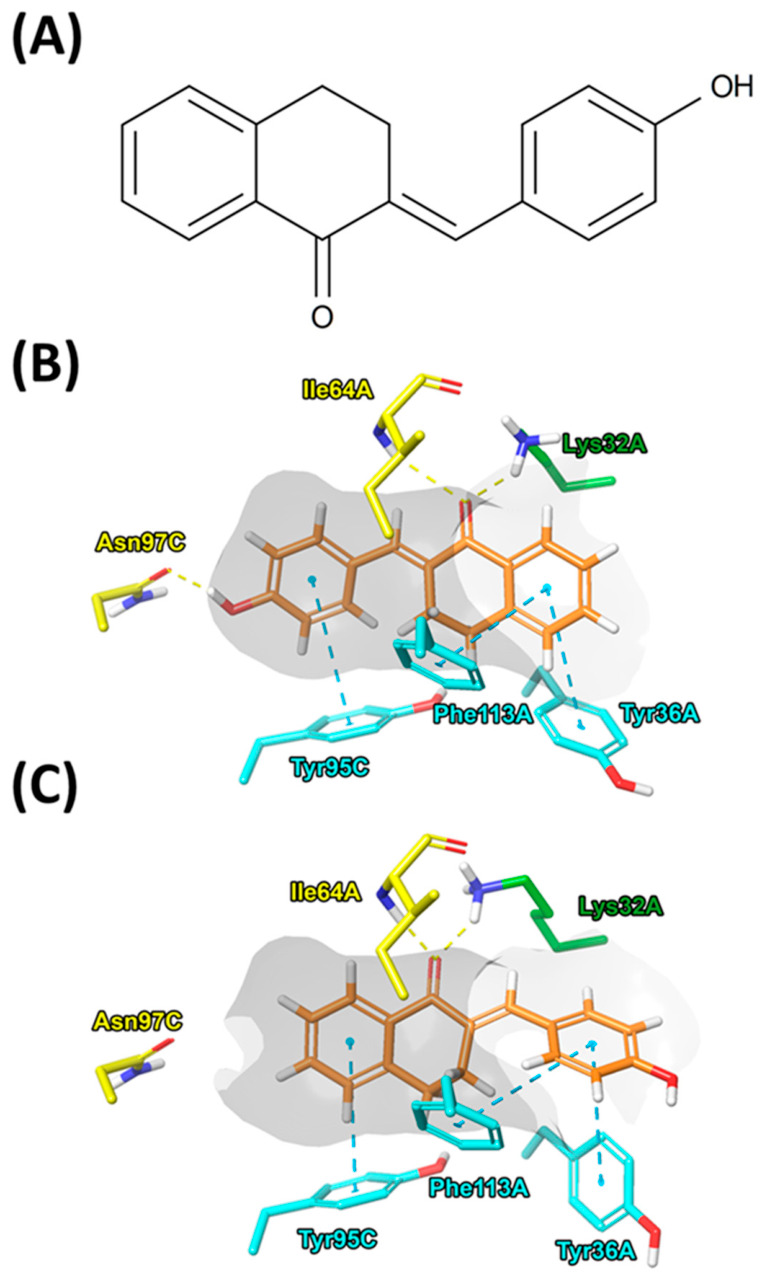
MIF tautomerase inhibitor TE-91: (**A**) Structural formula of (*E*)-6-Hydroxy-2-(4-hydroxybenzylidene)-3,4-dihydronaphthalene-1(2H)-one (TE-91). (**B**,**C**) Binding modes of TE-91 in the active site of MIF, identified through reversible docking experiments.

In accordance with the results published, two binding poses were predicted for TE-91 [20]. The residues involved are Ile64, Lys32, Tyr36, and Phe113 of one MIF chain, and Asn97 and Tyr95 of the adjacent chain. The docking score of the binding pose in which the 4-hydroxyphenyl moiety interacts with Asn97C is −8.182 kcal/mol (Figure 1B). In opposition to many other tetralone derivatives, the position of the hydroxyl group and subsequent interaction with the Asn97C residue of TE-91 enhances the binding affinity of this kind of orientation of the ligand. The docking score determined for the pose, where the 4-hydroxyphenyl moiety faces outwards MIF and is accessed by the solvent, is −7.961 kcal/mol (Figure 1C). In this pose, the interaction with Asn97C, often the most important for many MIF inhibitors, is absent. However, tetralone derivatives without hydroxyl substituents, such as TE-11, can modulate the activity of MIF. The docking results indicate that both binding orientations of TE-91 are possible.

### 3.2. Selective Enolase Inhibitor TE-91 Attenuates the Migration of Human Neutrophils Similarly to the Selective Ketonase Inhibitor KRP-6 and Counteracts MIF-Induced Anti-Apoptotic Effect

**Figure 2:**
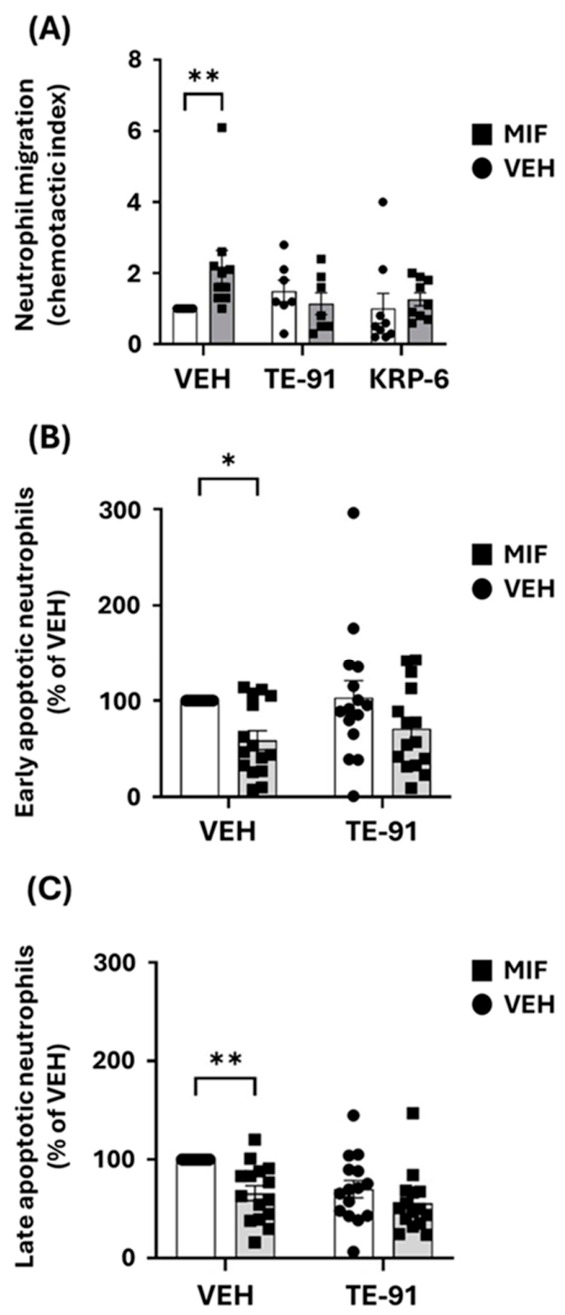
TE-91 prevents MIF-mediated migratory and anti-apoptotic responses in human neutrophils: (**A**) Purified polymorphonuclear leukocytes (PMNLs) were pretreated with 20 µM KRP-6 or 20 µM TE-91 for 30 min, then allowed to migrate toward MIF (3 nM) for 60 min in a micro-Boyden chamber at 37 °C. Migrated cells were quantified by flow cytometry and expressed as the chemotactic index (ratio of migrated cells in treated samples to baseline [vehicle control]). (**B**,**C**) PMNLs were treated with 20 µM TE-91 for 60 min in RPMI 160 medium supplemented with 3% FBS and 1% P/S at 37 °C. Cells were then stimulated with 500 nM MIF or vehicle (BSA in PBS). After 24 h, apoptosis was assessed by staining with APC–annexin V (1:100) and PI (1:50), followed by flow cytometric analysis. Data are presented as (**A**) mean ± SEM, combined data of n = 14–18 (7–9 independent experiments performed in technical duplicates), and (**B**,**C**) mean ± SEM as a percent of VEH-treated groups, n = 30 (15 independent experiments performed in technical duplicates). Statistical analysis was performed using (**A**) a mixed-effects model and (**B**,**C**) two-way ANOVA followed by Tukey’s post hoc test. * *p* < 0.05, ** *p* < 0.01. Effect sizes: (**A**) row factor: η2 = 0.11, column factor: η2 = 0.21, interaction: η2 = 0.42; (**B**) row factor: η2 = 0.010, column factor: η2 = 0.162, interaction: η2 = 0.004; (**C**): row factor: η2 = 0.128, column factor: η2 = 0.176, interaction: η2 = 0.035. Abbreviations: VEH: vehicle, MIF: macrophage migration inhibitory factor, BSA: bovine serum albumin, PBS: phosphate-buffered saline, APC: allophycocyanin, PI: propidium iodide, FBS: fetal bovine serum, P/S: penicillin/streptomycin.

Our results revealed MIF-induced neutrophil migrations (Figure 2). TE-91 and KRP-6 significantly reduced the MIF-induced migratory response in neutrophils (Figure 2A). MIF significantly attenuated early (Figure 2B) and late (Figure 2C) apoptosis in isolated human neutrophils, and TE-91 was capable of diminishing this anti-apoptotic effect.

### 3.3. TE-91 Attenuates Basal Extracellular Acidification Rate in RAW264.7 Macrophage Cells

**Figure 3:**
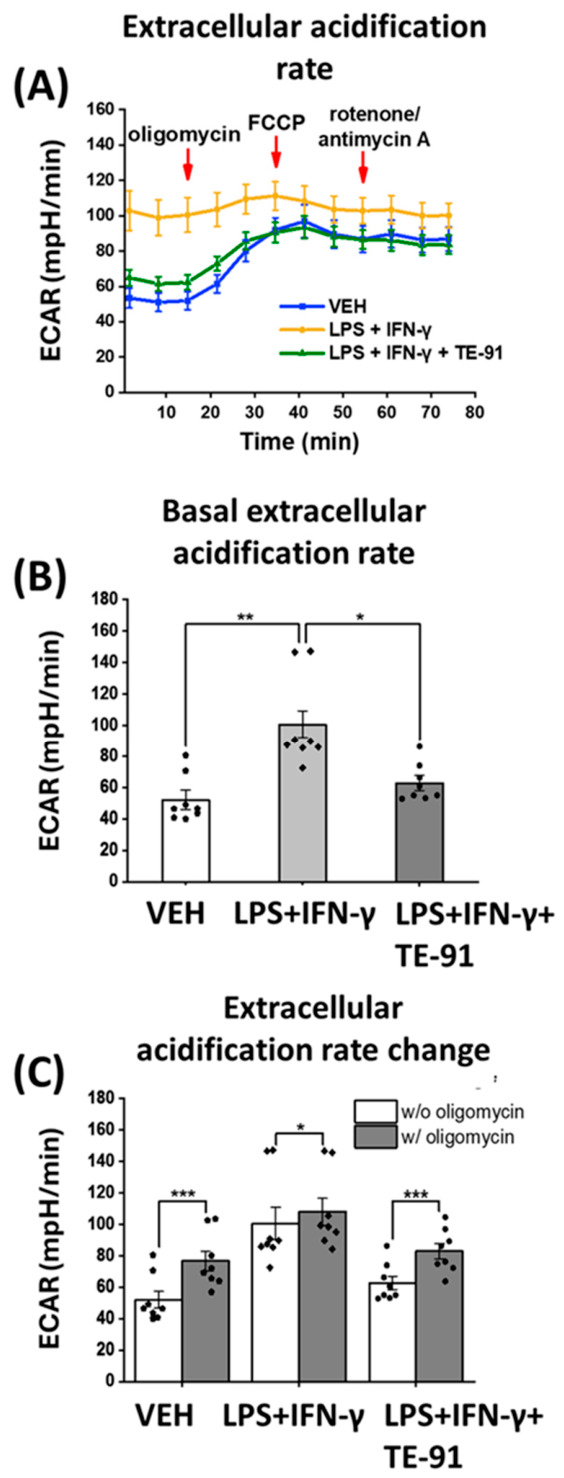
TE-91 attenuates aerobic glycolysis in activated macrophages: RAW264.7 cells were induced with 0.1 µg/mL LPS + 0.01 µg/mL IFN-γ after a 30 min pre-treatment with 20 µM TE-91. A total of 8 h later, the Seahorse XFp Mito Stress test was performed. DMSO was added to the VEH and LPS + IFN-γ groups in the same amount as TE-91-treated cells. Oligomycin, FCCP, and rotenone/antimycin A mixture were added during the measurement in a final concentration of 1 µM. (**A**) ECAR, (**B**) basal ECAR, and (**C**) ECAR change before and after oligomycin injection are shown. Data are represented as mean ± SEM, combined data of n = 8 (4 independent experiments with 2 biological replicates). Statistical analyses were performed by (**B**) Welch’s ANOVA and (**C**) Student’s paired dependent *t*-test. * *p* < 0.05, ** *p* < 0.01, *** *p* < 0.001. Effect sizes: (**B**): η2 = 0.551, and (**C**): VEH: d = 4.164, LPS + IFN-γ: d = 1.130, LPS + IFN-γ + TE-91: d = 2.538. Abbreviations: ECAR: extracellular acidification rate, VEH: vehicle, LPS: lipopolysaccharide, IFN-γ: interferon-γ, FCCP: carbonyl cyanide-4(trifluoromethoxy)-phenylhydrazone.

Glycolytic activity was assessed by determining ECAR in activated RAW264.7 cells. Our results revealed that LPS + IFN-γ increased basal ECAR in macrophages when compared to VEH-treated cells, while TE-91 diminished ECAR in comparison to the LPS + IFN-γ-activated group (Figure 3A [1–3 points of the measurement] and Figure 3B). Oligomycin increased ECAR in all treatment groups; however, it had the most moderate effect in the LPS + IFN-γ group (Figure 3A [4–6 points of the measurement] and Figure 3C). In our model, FCCP (Figure 3A [7–9 points of the measurement]) and rotenone with antimycin A did not substantially influence ECAR (Figure 3A [10–12 points of the measurement]).

### 3.4. TE-91 Improves Mitochondrial Respiration, ATP Synthesis, and Spare Respiratory Capacity in Activated Macrophages

**Figure 4:**
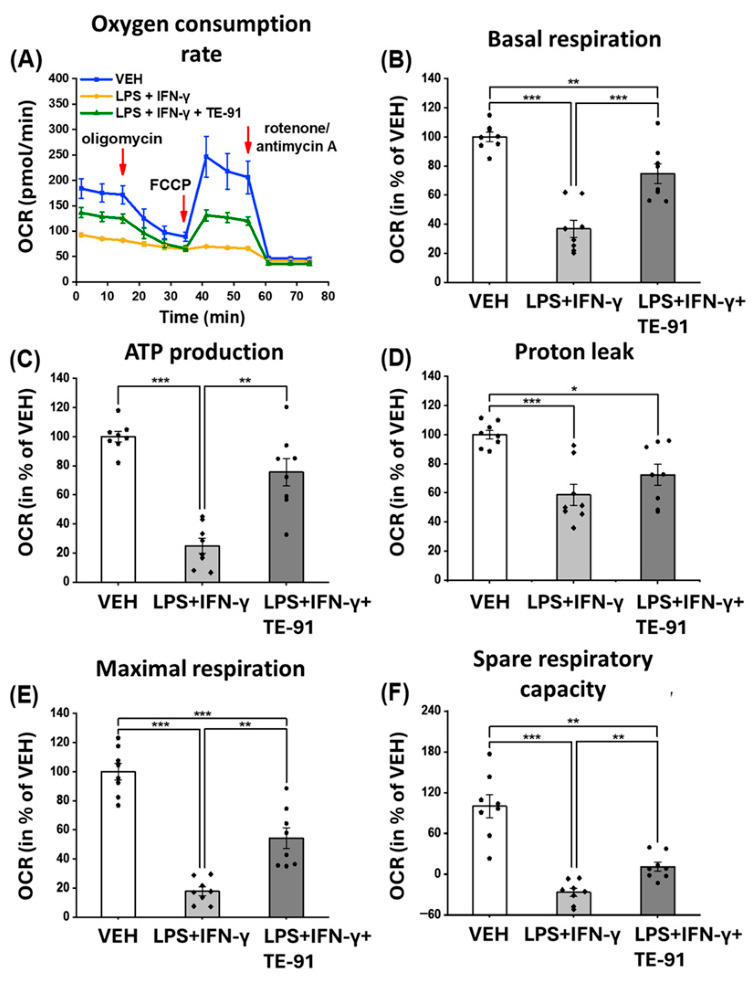
TE-91 preserves mitochondrial respiration and enhances mitochondrial bioenergetic parameters in LPS + IFN-γ-induced macrophages: RAW264.7 cells were pre-treated with 20 µM TE-91 for 30 min and then induced with 0.1 µg/mL LPS + 0.01 µg/mL IFN-γ for 8 h. VEH- and LPS + IFN-γ-treated groups received the same amount of DMSO as TE-91-treated cells. Seahorse XFp Mito Stress test was carried out, where a 1 µM final concentration of oligomycin, FCCP, and rotenone/antimycin A mixture was added, respectively. (**A**) OCR, (**B**) basal respiration, (**C**) ATP production, (**D**) proton leak, (**E**) maximal respiration, and (**F**) spare respiratory capacity are shown as mean ± SEM (**B**–**F**) as a percent of VEH. Statistical analyses were carried out by (**B**,**D**–**F**) one-way ANOVA, and (**C**) Welch’s ANOVA. n = 8 (combined data of 4 independent experiments with 2 biological replicates). * *p* < 0.05, ** *p* < 0.01, *** *p* < 0.001. Effect sizes: (**B**): η2 = 0.766, (**C**): η2 = 0.763, (**D**): η2 = 0.527, (**E**): η2 = 0.839, (**F**): η2 = 0.770. Abbreviations: OCR: oxygen consumption rate, VEH: vehicle, LPS: lipopolysaccharide, IFN-γ: interferon-γ, FCCP: carbonyl cyanide-4(trifluoromethoxy)-phenylhydrazone.

Mitochondrial function was analyzed by evaluating OCR in M1 polarized RAW264.7 cells (Figure 4A,B). By using specific inhibitors of the mitochondrial respiratory chain complexes, such as oligomycin, FCCP, and rotenone plus antimycin A, further mitochondrial bioenergetic parameters were determined and calculated (Figure 4A–F). We discovered LPS + IFN-γ reduced basal respiration in macrophages in comparison to VEH cells (Figure 4A [1–3 points of the measurement], and Figure 4B). Moreover, oligomycin attenuated respiration (Figure 4A [4–6 points of the measurement]), while FCCP (Figure 4A [7–9 points of the measurement]) increased basal respiration in VEH- and LPS + IFN-γ + TE-91-treated cells. However, in LPS + IFN-γ-treated macrophages, both oligomycin and FCCP failed to modulate cellular respiration. LPS + IFN-γ strongly decreased ATP production (Figure 4C), proton leakage (Figure 4D), maximal respiration (Figure 4E), and spare respiratory capacity (Figure 4F); hence, TE-91 successfully counteracted all investigated parameters except for proton leakage.

### 3.5. TE-91 Restores Aerobic, Energetic Metabolism in Activated Macrophages

**Figure 5:**
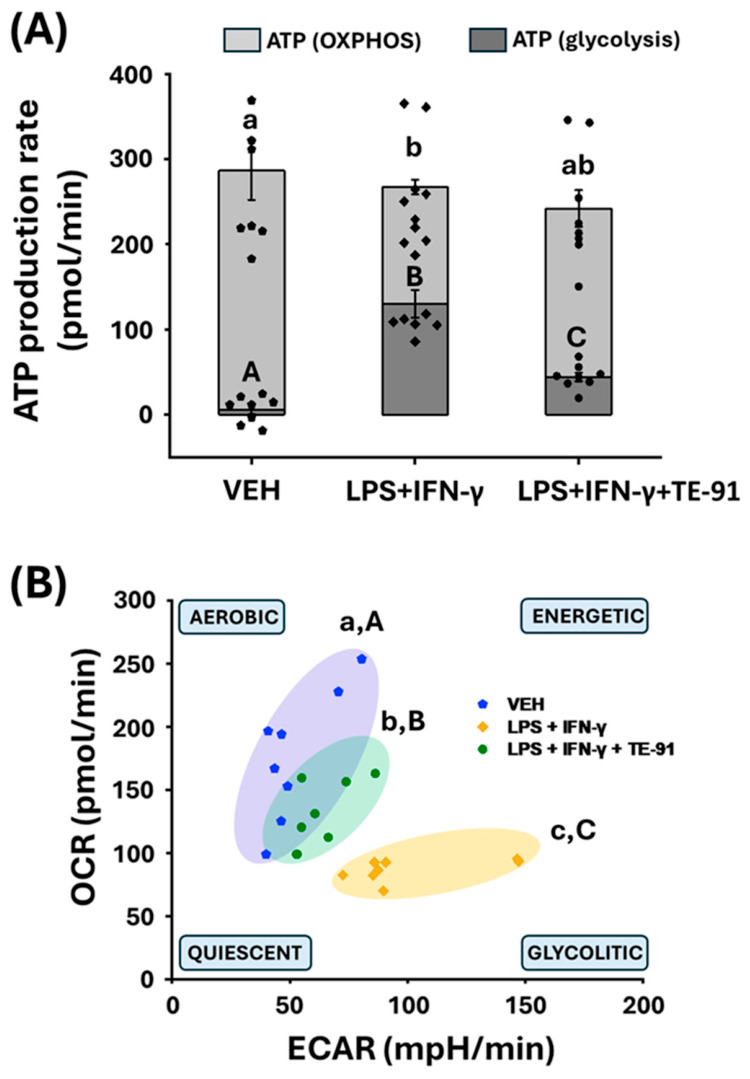
TE-91 restores aerobic, energetic cell metabolism in M1 macrophages: RAW264.7 cells were pre-treated with 20 µM TE-91 for 30 min and then induced with 0.1 µg/mL LPS + 0.01 µg/mL IFN-γ for 8 h. VEH- and LPS + IFN-γ-treated groups received the same amount of DMSO as TE-91-treated cells. Seahorse XFp Mito Stress test was carried out, where a 1 µM final concentration of oligomycin, FCCP, and rotenone/antimycin A mixture was added, respectively. (**A**) Calculated ATP production rate in OXPHOS and glycolysis, and (**B**) energy map for macrophage cells is shown, n = 8 (combined data of 4 independent experiments with 2 biological replicates). Statistical analyses were performed with (**A**,**B**): one-way ANOVA with Games–Howell pairwise comparison. Means for a variable without a common letter differ (*p* < 0.05). Lowercase letter was used for (a): OXPHOS, (b,c): OCR, and capital letter was used for (A): glycolysis, (B,C): ECAR. Effect sizes: (**A**): ATP (OXPHOS): η2 = 0.464, ATP (glycolysis): η2 = 0.783, and (**B**): OCR: η2 = 0.574, ECAR: η2 = 0.551. Abbreviations: VEH: vehicle, LPS: lipopolysaccharide, IFN-γ: interferon-γ, FCCP: carbonyl cyanide-4(trifluoromethoxy)-phenylhydrazone, OCR: oxygen consumption rate, ECAR: extracellular acidification rate, OXPHOS: oxidative phosphorylation.

LPS + IFN-γ treatment induced a strong metabolic shift in M1 macrophages from oxidative phosphorylation (OXPHOS) to glycolysis, with significantly lower mitochondrial/glycolytic ATP production ratio (Figure 5A) compared to VEH-treated cells (Figure 5B). TE-91 reversed this metabolic shift and induced a more energetic and aerobic metabolism, and improved mitochondrial/glycolytic ATP production ratio (Figure 5B).

### 3.6. TE-91 Reduces Inflammatory Response in Activated Macrophages at mRNA Level

**Figure 6:**
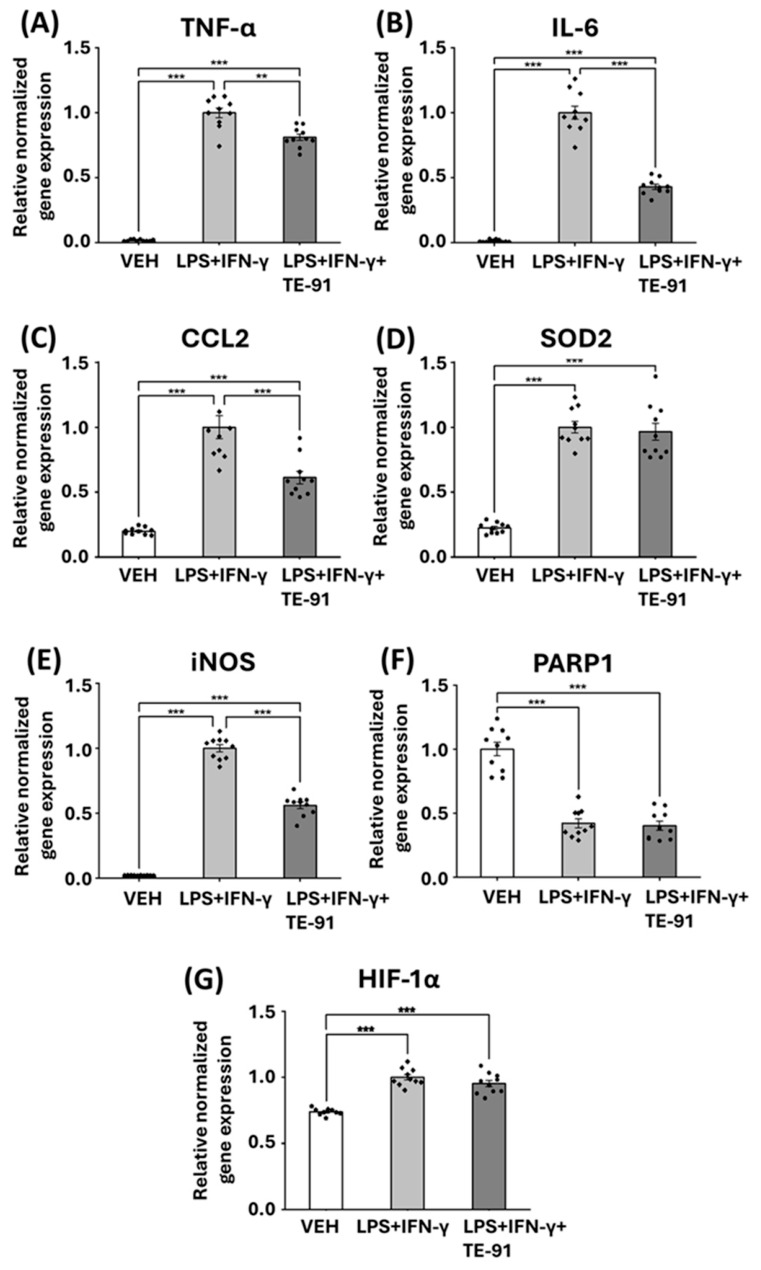
TE-91 modulates inflammatory mRNA transcription in macrophages: RAW264.7 cells were induced with 0.1 µg/mL LPS + 0.01 µg/mL IFN-γ following a 30 min pre-treatment with 20 µM TE-91. Relative normalized gene expressions of (**A**) TNF-α, (**B**) IL-6, (**C**) CCL2, (**D**) SOD2, (**E**) iNOS, (**F**) PARP1, and (**G**) HIF-1α are shown. Combined data of n = 10 (5 independent experiments with two biological replicates) is represented as mean ± SEM as a percent of LPS + IFN-γ-treated groups. Statistical analyses were carried out by (**A**) one-way ANOVA and (**B**–**G**) Welch’s ANOVA. ** *p* < 0.01, *** *p* < 0.001. Effect sizes: (**A**): η2 = 0.968, (**B**): η2 = 0.949, (**C**): η2 = 0.781, (**D**): η2 = 0.867, (**E**): η2 = 0.975, (**F**): η2 = 0.829, (**G**): η2 = 0.762. Abbreviations: VEH: vehicle, LPS: lipopolysaccharide, IFN-γ: interferon-γ, TNF-α: tumor necrosis factor α, IL-6: interleukin 6, CCL2: C-C motif chemokine ligand 2, SOD2: superoxide dismutase 2, iNOS: inducible nitric oxide synthase, PARP1: poly(ADP-ribose) polymerase 1, HIF-1α: hypoxia inducible factor 1, alpha subunit.

LPS + IFN-γ enhanced the mRNA levels of TNF-α (Figure 6A), IL-6 (Figure 6B), CCL2 (Figure 6C), superoxide dismutase 2 (SOD2, Figure 6D), inducible nitric oxide synthase (iNOS, Figure 6E), and HIF-1α (Figure 6G) while downregulating poly(ADP-ribose) polymerase 1 (PARP1) mRNA (Figure 6F) in macrophages. We also discovered MIF enolase inhibitor TE-91 significantly attenuated TNF-α (Figure 6A), IL-6 (Figure 6B), CCL2 (Figure 6C), and iNOS (Figure 6E), yet failed to influence SOD2 (Figure 6D), PARP1 (Figure 6F), and HIF-1α (Figure 6G) mRNA levels when compared to the LPS + IFN-γ treatment group.

### 3.7. TE-91 Reduces RONS and Inflammatory Cytokine Production in M1 Macrophages

**Figure 7:**
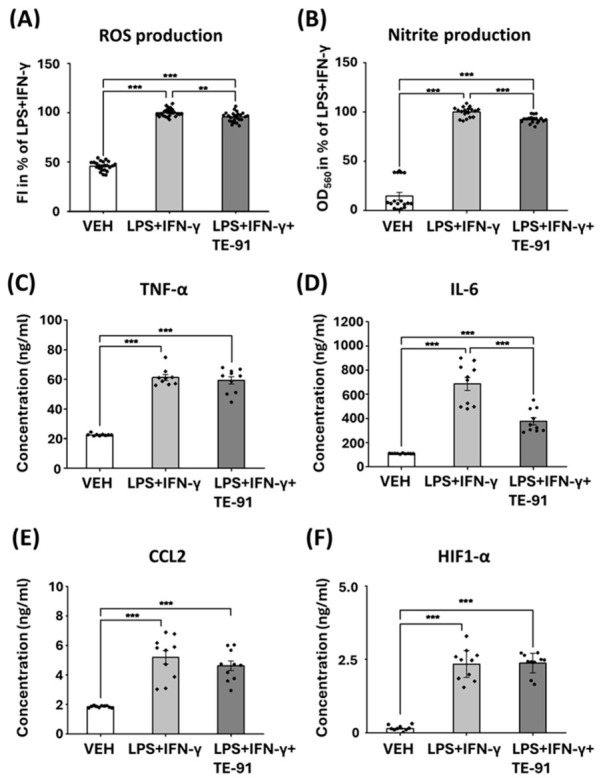
TE-91 inhibited ROS and nitrite production, and IL-6 and CCL2 expression: RAW264.7 cells were pre-treated with 20 µM TE-91 for 30 min prior to 0.1 µg/mL LPS and 0.01 µg/mL IFN-γ treatment for another 24 h. (**A**) ROS production was detected by adding 2 µM DHR123 fluorescent dye (490 nm excitation/ 510–570 nm emission). (**B**) Nitrite production was determined by mixing equal amounts of supernatant and Griess–Ilosvay reagent, optical density was measured at 560 nm. (**C**–**F**) TNF-α, IL-6, CCL2, and HIF-1α concentrations were measured from supernatant via ELISA kits. Data are presented as mean ± SEM as a percent of LPS + IFN-γ-treated groups, combined data of (**A**) n = 24 (4 independent experiments with six biological replicates), (**B**) n = 18 (three independent experiments with six biological replicates), and (**C**–**F**) n = 10 (five independent experiments with two biological replicates). Statistical analyses were performed via (**A**,**D**) one-way ANOVA, and (**B**,**C**,**E**,**F**) Welch’s ANOVA. ** *p* < 0.01, *** *p* < 0.001. Effect sizes: (**A**): η2 = 0.968, (**B**): η2 = 0.943, (**C**): η2 = 0.870, (**D**): η2 = 0.829, (**E**): η2 = 0.692, and (**F**): η2 = 0.892. Abbreviations: VEH: vehicle, LPS: lipopolysaccharide, IFN-γ: interferon-γ, ROS: reactive oxygen species, TNF-α: tumor necrosis factor-α, IL-6: interleukin-6, CCL2: C-C motif chemokine ligand-2, HIF-1α: hypoxia inducible factor 1, alpha subunit, DHR123: dihydrorhodamine 123, FI: fluorescent intensity, OD: optical density.

LPS + IFN-γ induced ROS (Figure 7A) and nitrite (Figure 7B) production, accompanied by TNF-α (Figure 7C), IL-6 (Figure 7D), CCL2 (Figure 7E), and HIF-1α (Figure 7F) protein expression. TE-91 slightly reduced ROS (Figure 7A), nitrite (Figure 7B), and IL-6 (Figure 7D) production and failed to modulate TNF-α (Figure 7C), CCL2 (Figure 7E), and HIF-1α (Figure 7F) in activated macrophage cells.

### 3.8. TE-91 Decreases the Expression of Cleaved IL-1β but Does Not Modulate Pro-IL-1β Expression

**Figure 8:**
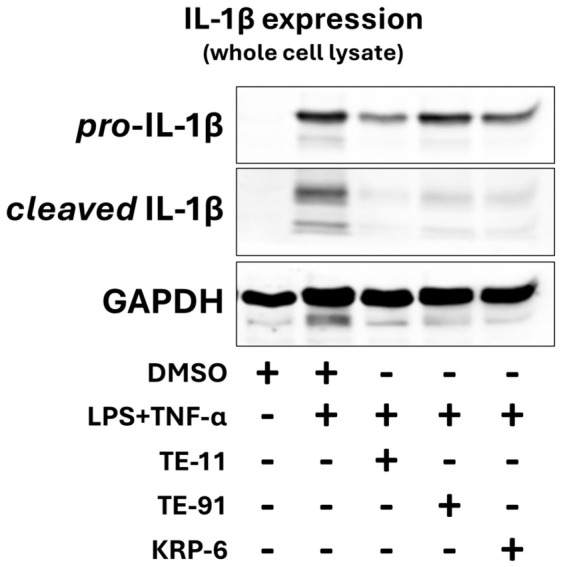
Effect of various MIF tautomerase inhibitors on IL-1β expression: Raw264.7 cells were induced with 0.1 µg/mL LPS and 1 µg/mL mouse TNF-α for 24 h following a 30 min pretreatment with 20 µM TE-91, TE-11, or KRP-6. Cell lysates were collected, protein concentration quantified, and samples were immunoblotted for IL-1β and GAPDH. Abbreviations: IL-1β: interleukin-1β, GAPDH: glyceraldehyde 3-phosphate dehydrogenase, DMSO: dimethyl sulfoxide, LPS: lipopolysaccharide, TNF-α: tumor necrosis factor-α.

LPS + TNF-α induced a marked increase in pro-IL-1β expression and strongly enhanced the amount of cleaved, active form of IL-1β (Figure 8). TE-11 and KRP-6 attenuated both the concentration of pro-IL-1β and cleaved IL-1β, while TE-91 decreased IL-1β and did not modify pro-IL-1β levels (Figure 8). Full-length western blot images for pro-IL-1β and cleaved-IL1β are shown as Supplementary Materials (Supplementary Figure S1).

### 3.9. TE-91 Acts as a Radical Scavenger in Cell-Free Systems but the Vehicle DMSO Masks This Effect

**Figure 9:**
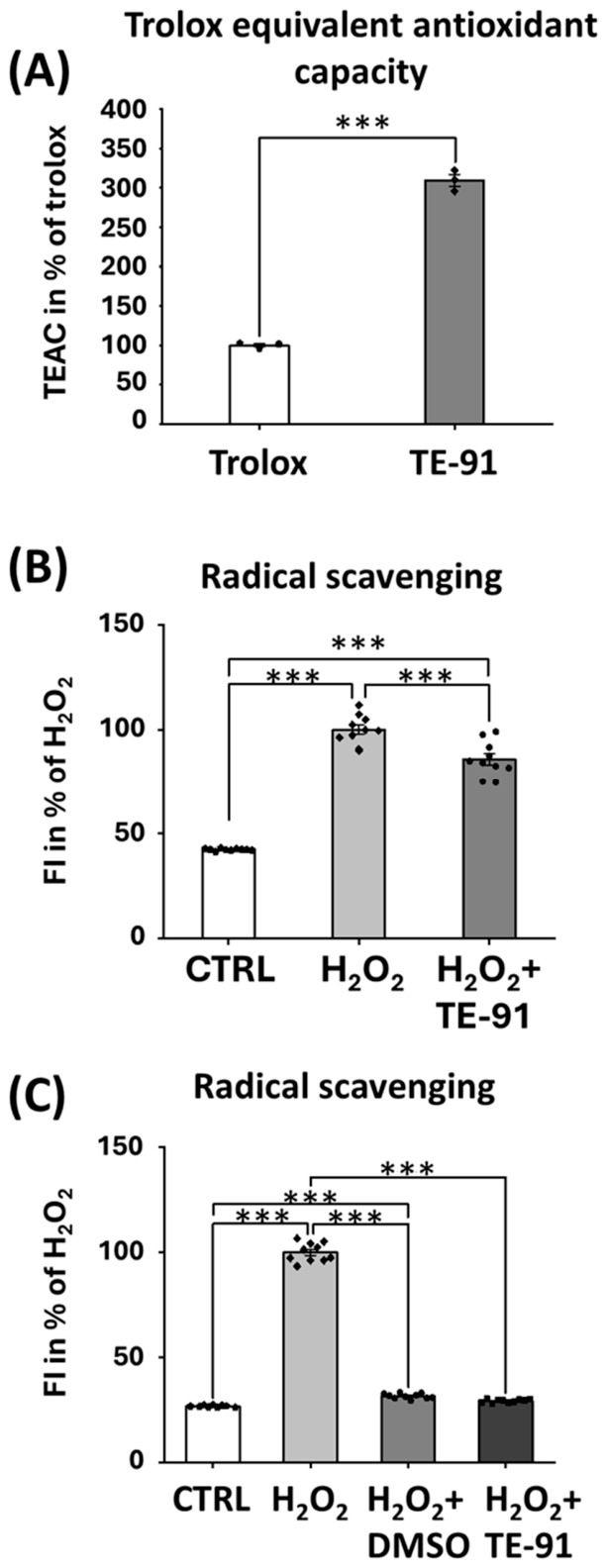
TE-91 is a radical scavenger in cell-free systems, but the vehicle DMSO totally masks this effect: (**A**) TEAC assay was performed by incubating TE-91 or Trolox with ABTS^•+^ radical cation, and the percentage inhibition of absorbance was measured (OD_734_ nm). (**B**,**C**) Peroxide radical concentrations were measured in a cell-free system by adding 100 µM H_2_O_2_ and 100 µM EDTA-Fe^2+^ salt to PBS with (**B**) 20 µM TE-91 in PBS and (**C**) DMSO alone or 20 µM TE-91 in DMSO. Radical scavenging activity was detected by adding 2 µM DHR123 fluorescent dye (490 nm excitation/510–570 nm emission). Data are represented as mean ± SEM (**A**) as a percent of Trolox, (**B**,**C**) as a percent of H_2_O_2_, combined data of (**A**) n = 3 (five independent concentration measurements repeated three times), (**B**,**C**) n = 10 (ten independent experiments). Statistical analyses were performed via (**A**) independent samples *t*-test, and (**B**,**C**) one-way ANOVA. *** *p* < 0.001. Effect sizes: (**A**): d = 21.665, (**B**): η2 = 0.945, (**C**): η2 = 0.995. Abbreviations: TEAC: Trolox equivalent antioxidant capacity, ABTS^•+^: 2,2′-azino-bis(3-ethylbenzothiazoline-6-sulfonic acid), CTRL: control, DHR123: dihydrorhodamine 123, FI: fluorescent intensity, OD: optical density.

TE-91 significantly attenuated the concentration of ABTS^•+^ (Figure 9A) and OH^•^ (Figure 9B) radicals. However, DMSO, which was utilized as a vehicle in our cell culture experiments, completely masked the radical scavenging activity of TE-91 in the Fenton reaction (Figure 9C).

### 3.10. TE-91 May Have Lower Capability to Pass Cell Membranes in Comparison to TE-11 and KRP-6

In our performed experiments, physicochemical and ADME properties of TE-91, TE-11, and KRP-6 were predicted and underwent comparison. As predicted by the structures, TE-91 was determined as the least lipophilic compound with the largest polar surface area (Table 2). Therefore, TE-91 was expected to have a lower capability to pass cell membranes when compared to KRP-6 and TE-11. This hypothesis was supported by the predicted Caco-2 and MDCK permeability values (Table 2). This difference in polarity may account for the different effects exerted by the analogs.

### 3.11. TE-91 Reduces ROS, Nitrite, and IL-6 Production in PM and in BMDM Cells

To confirm some of our results in primary macrophage cells, we examined LPS + IFN-γ-induced ROS and nitrite production along with TNF-α and IL-6 protein expression in PM (Supplementary Figure S2) and in BMDM cells (Supplementary Figure S3). LPS + IFN-γ enhanced all the measured parameters, both in PMs and in BMDMs. TE-91, similarly to RAW264.7 cells, reduced ROS (Supplementary Figures S2A and S3A), nitrite (Supplementary Figures S2B and S3B), and IL-6 production (Supplementary Figures S2D and S3D), but failed to modulate TNF-α (Supplementary Figures S2C and S3C), both in PM and BMDM cells.

## 4. Discussion

In our present study, we revealed that TE-91, a selective MIF enolase inhibitor, reduces M1 polarization with associated inflammatory metabolic reprogramming in RAW264.7 macrophage cells. We also confirmed some of our results in PM and BMDM cells. Furthermore, we unveiled several differences between the anti-inflammatory effects of TE-91 and the previously described highly selective ketonase inhibitor KRP-6 [19,21]. We also applied the non-selective inhibitor TE-11 [19,22], which effectively inhibits both the ketonase and enolase sub-activities.

Previously, we showed that tetralone and indanone derivatives directly bind to the active site of MIF and inhibit its tautomerase enzyme activity [19,20]. We also confirmed MIF’s action as a cytokine is performed by binding its specific receptors, such as CD74 [29] or the MIF co-receptors CXCR2 and CXCR4 [30]. However, receptor binding and activation were mediated by the tautomerase active site [31]. Thus, our first aim was to demonstrate the binding of TE-91 (Figure 1A) to the tautomerase active site of MIF by performing molecular docking analyses in silico (Figure 1B,C). Reversible docking studies suggested TE-91 may bind directly to MIF in the tautomerase active site in two different binding poses (Figure 1B,C). Since molecular docking analyses only predict the binding of a compound, our next aim was to demonstrate its physical binding to MIF indirectly in a biological system.

Therefore, we examined MIF-induced leukocyte migration and apoptosis (Figure 2), which are both linked to receptor binding and activation [30,32]. In our leukocyte migration experiments, we also utilized the selective ketonase inhibitor KRP-6 [19,21] and compared its effect with that of the selective enolase inhibitor TE-91. We found KRP-6 and TE-91 equally inhibited MIF-induced neutrophil (Figure 2A) chemotaxis. We discussed the effect of MIF inhibition in the same model in more detail in our previous studies [21,22]. However, our present results revealed novel conclusions previously missing. Namely, both TE-91, the selective enolase inhibitor, and KRP-6, the selective ketonase inhibitor, prevented MIF receptor activation equally (Figure 2A), as also did the non-selective tautomerase inhibitor TE-11 [22]. Thus, we conclude that regardless of selectivity, all types of tautomerase inhibitors that bind the active site of MIF do prevent MIF’s binding to its receptors. Since MIF-induced leukocyte survival is equally mediated by MIF receptor activation [33], the fact that TE-91, similar to KRP-6 [21] and TE-11 [22], prevented neutrophil survival further supported this notion (Figure 2B,C). Therefore, we hypothesized that all pro-inflammatory effects of MIF as a cytokine can be equally suppressed by utilizing both types of tautomerase inhibitors.

Accordingly, we examined proinflammatory processes mediated, at least partially, by MIF receptor activation, with a focus on metabolic reprogramming. MIF appears to broadly regulate metabolic reprogramming in cells. For example, hypoxia-induced metabolic reprogramming is mediated by a MIF/IL-6/JAK-STAT signaling axis, which affects lipid metabolism in laryngocarcinoma cells [34]. In pancreatic ductal adenocarcinoma cells, a MIF-driven signaling pathway inhibits nuclear receptor subfamily 3 group C member 2 (NR3C2) transcription factor activation. The signal transduction process involves the CD74 receptor and activates the PI3K/Akt pathway, which mediates the production of the miR-301b microRNA. In turn, miR-301b inhibits NR3C2 activation [35]. This MIF/NR3C2 axis further regulates the MAPK/ERK and AP-1 pathways, thereby controlling glycolytic reprogramming of pancreatic cancer cells [36]. MIF also controls glycolytic switch in breast cancer cells by stimulating the WNT/β-catenin pathway, thereby increasing c-MYC-dependent transcription of aldolase C, a glycolytic enzyme [37]. Therefore, we investigated the processes of glycolysis in our M1 macrophage model. In these experiments, TE-91 prevented LPS + IFN-γ-induced proinflammatory metabolic switch, which enhances basal ECAR (Figure 3A,B), a principal representative of aerobic glycolysis [24]. We found that increased glycolytic flux nearly reached its plateau in LPS + IFN-γ-treated macrophages, since neither the F_O_F_1_-ATPase inhibitor oligomycin, nor the mitochondrial uncoupler FCCP, nor the respiratory chain complex I inhibitors rotenone and antimycin A could induce a marked increase in ECAR (Figure 3A,C). In contrast, oligomycin can significantly increase ECAR in VEH- and in LPS + IFN-γ + TE-91-treated cells (Figure 3A,C). Moreover, lower basal ECAR (Figure 3A,B) and the improving effect of oligomycin in LPS + IFN-γ + TE-91-treated cells (Figure 3A,C) undoubtedly denoted suppressed glycolytic activity following TE-91 treatment. This reduction in glycolytic rate definitely leads to the inhibition of inflammatory M1 activation in macrophages. In contrast, TE-91 significantly enhanced OCR in LPS + IFN-γ-treated cells (Figure 4A), causing an improved basal respiration (Figure 4B), maximal respiration (Figure 4E), spare respiratory capacity (Figure 4F), and ATP production (Figure 4C). This implies TE-91 may prevent a strong reduction in OXPHOS, which, together with the inhibition of glycolysis, diminishes M1-associated metabolic reprogramming in macrophage cells. Thus, TE-91 restores a more energetic, aerobic metabolism of inactivated cells (Figure 5). Thus, TE-91 acted in a very similar manner as the highly selective MIF ketonase inhibitor KRP-6 and the non-selective MIF tautomerase inhibitor TE-11 from our previous studies [21,22]. All these results strongly suggest MIF-induced metabolic reprogramming is fundamentally mediated by MIF’s receptor binding and activation, regardless of its tautomerase sub-activities.

Next, we equally investigated alterations in mRNA transcription, protein expression, transcription factor activation, and RONS production associated with M1 activation. We discovered TE-91 inhibited TNF-α, IL-6, CCL2, and iNOS mRNA production in activated macrophages, yet failed to modulate SOD2 and PARP1 transcription (Figure 6). Interestingly, the selective ketonase inhibitor KRP-6 did, however, only slightly increase PARP1 mRNA transcription in our previous study utilizing the same model [21]. More interestingly, the non-selective tautomerase inhibitor TE-11, which also has a strong ketonase inhibiting activity, significantly improved SOD2 transcription [22]. Knowingly, HIF-1α directly binds to the hypoxia response element of the SOD2 promoter, thereby inhibiting SOD2 gene expression [38]. In our previous study, TE-11 inhibited HIF-1α mRNA transcription and protein expression in the same model; thus, we hypothesized that reduced HIF-1α activation prevents the blockade of SOD2 mRNA transcription [22]. Accordingly, we determined the effect of TE-91 on HIF-1α. Surprisingly, TE-91 did not inhibit HIF-1α mRNA transcription (Figure 6G) or protein expression (Figure 7F), which may clearly explain why TE-91 failed to enhance SOD2 mRNA transcription. Thus, we revealed the first significant distinction between the effects of TE-91 and TE-11. One obvious explanation may be that TE-11 is non-selective and also inhibits the ketonase activity of MIF, while TE-91 only inhibits its enolase activity. It has been previously reported that the ketonase sub-activity of isolated MIF is more pronounced than its enolase activity [39]. This may suggest the ketonase activity of MIF is most likely also pronounced in cells, indicating that MIF itself functions primarily as a ketonase.

Interestingly, we also observed some discrepancies in the analysis of mRNA transcription and protein translation. The mRNA transcription of TNF-α and CCL2 was inhibited by TE-91, but TE-91 failed to modulate their protein levels (Figure 6A,C and Figure 7C,E). One possible reason for these discrepancies could be the shedding of cytokines. For example, TNF-α mRNA is very short-lived, even in M1 macrophages [40], so the inhibition of transcription rapidly reduces mRNA concentration. In contrast, LPS can activate tumor necrosis factor-α-converting enzyme, which induces TNF-α shedding and thus secretion into culturing medium [41] in which TNF-α protein is quite stable even 24 h after induction [42]. Since our measurements were performed in cell culture medium of treated cells and not on cell extracts, the large amount of previously secreted cytokines may mask the effect of transcriptional regulation.

Previously, we concluded that regardless of the selectivity of inhibitors, similar effects of TE-11 and TE-91 are likely due to the inhibition of MIF’s receptor binding. However, the significant differences between the two compounds may suggest MIF-ketonase sub-activity is essential for MIF-induced receptor-independent mechanisms. One possible receptor-independent mechanism is the release of IL-1β, in which MIF knowingly plays a critical role [43]. It has been shown that MIF can influence IL-1β release through activation of the NLR family pyrin domain-containing 3 (NLRP3) inflammasome, in which MIF was essential for the NLRP3/vimentin interaction, and it directly binds to NLRP3. Again, this regulating role of MIF in inflammasome activation is entirely independent of receptor activation [43]. Thus, we investigated pro-IL-1β expression and also the cleaved, active form of IL-1β in LPS + TNF-α-activated macrophages, since RAW264.7 cells cannot release IL-1β upon LPS + IFN-γ treatment [44]. We found that the inhibitors TE-11 and KRP-6, which effectively reduce ketonase sub-activity, inhibited IL-1β production and activation. Whereas TE-91 did not modify pro-IL-1β concentration and modestly reduced its proteolytic cleavage in comparison with the two ketonase inhibitors (Figure 8). These findings may emphasize the role of MIF tautomerase in receptor-independent transcriptional processes and underline the importance of its ketonase sub-activity.

On the other hand, an alternative explanation for the inhibitors’ diverse effects suggests TE-91 may inhibit extracellular receptor binding only (i.e., the cytokine activity of MIF) yet may not strongly impact intracellular, receptor-independent mechanisms. Since we did not perform direct receptor blocking experiments, i.e., CD74/CXCR2/CXCR4 inhibition, this latter suggestion is a hypothesis. We can only assume that can occur, for example, if TE-91 crosses the cell membrane less efficiently than TE-11 or KRP-6. Thus, we investigated physicochemical properties of the three compounds. The analysis revealed a clear difference in polar surface area, in predicted apparent Caco-2 cell permeability, and in predicted apparent MDCK cell permeability (Table 2). These predictions imply TE-91 is a more polar molecule than TE-11 and KRP-6; thus, it is likely to be less readily taken up by macrophages. However, it must be stated that TE-91 can also enter the cells in reasonable amounts, which is clearly shown by the reduced concentration of cleaved IL-1β (Figure 8), since cleavage occurs intracellularly. In the absence of intracellular MIF activity or localization analysis, we can only hypothesize that the predicted physicochemical parameters and perhaps inhibitor selectivity both may account for the observed differences regarding the biological activity of the investigated inhibitors.

Finally, a third explanation for the discrepancies may be TE-91 exhibits a pro-oxidant activity, since ROS activates and antioxidants inhibit IL-1β release in M1-activated macrophages [45,46]. Accordingly, we hypothesized that mild direct pro-oxidant activity may reduce the more pronounced indirect antioxidant effects of MIF inhibition. In contrast, we found TE-91 directly scavenged ROS, which was demonstrated in cell-free systems such as Trolox-Equivalent Antioxidant Capacity (Figure 9A) or in the Fenton reaction (Figure 9B). The antioxidant effect of TE-91 in the Fenton reaction, however, was completely masked by DMSO (Figure 9C), the vehicle of TE-91, which raises the possibility of a significant vehicle effect. In addition to using vehicle control to exclude possible effects of DMSO in our model, the 0.2% concentration we used was unlikely to affect ROS production, since concentrations higher than 0.5% were the first to show significant antioxidant effects in RAW264.7 cells [47]. Thus, probably a combination of direct radical scavenging and an indirect antioxidant effect through MIF inhibition leads to the observed reduced ROS production in macrophages (Figure 7A).

We could also confirm that TE-91 had a very similar effect on isolated macrophage cells as on RAW264.7 cells (Supplementary Figures S1 and S2).

Comprehensively, we discovered TE-91 inhibits M1 macrophage activation with associated metabolic reprogramming by preventing MIF receptor activation. However, TE-91 revealed less inhibitory to the examined receptor-independent processes, which likely occurred due to its lower MIF ketonase sub-activity, or its less efficient cell-entry compared to TE-11 or KRP-6. Since we did not investigate selective enolase inhibitors other than TE-91, confirmation of the former hypothesis needs further investigation. In addition, it may equally raise the question of the relevance of tautomerase sub-activities in these processes.

## Figures and Tables

**Table 1:** The list of primers for gene expression analysis.

Name	Accession NM	Sequence (5′-3′) (F-Forward, R-Reverse)	Amplicon Size (bp)
TNF-α [tumor necrosis factor-α]	NM_001278601	F-ATGAGCACAGAAAGCATGATC	660
		R-TCACAGAGCAATGACTCCAA	
IL-6 [interleukin-6]	NM_031168	F-AGCCAGAGTCCTTCAGAGAGAT	108
		R-AGGAGAGCATTGGAAATTGGGG	
CCL2 [C-C motif chemokine ligand 2]	NM_011333	F-CTCAGCCAGATGCAGTTAACG	157
		R-CAGACCTCTCTCTTGAGCTTGG	
SOD2 [superoxide dismutase 2]	NM_013671	F-GGAGCAAGGTCGCTTACAGA	74
		R-GCGGAATAAGGCCTGTTGTT	
iNOS [inducible nitric oxide synthase]	NM_010927	F-GGGCAGCCTGTGAGACCTT	72
		R-CATTGGAAGTGAAGCGTTTCG	
PARP1 [poly(ADP-ribose) polymerase 1]	NM_007415	F-GAGTACAGTGCCAGTCAGC	117
		R-CACCTCGTCACCTTTTCTCTT	
HIF-1α [hypoxia inducible factor 1, alpha subunit]	NM_001422143	F-TTAGCATGCAGACTGCTGGG	102
		R-GTTTCTGCTGCCTTGTATGGG	
RPL27 [ribosomal protein L27]	NM_011289	F-AGGTCAAGTTTGAGGAGCGATAC	141
		R-CCCACACAAATGCAATAGGCAG	

**Table 2:** Physicochemical properties of TE-91, TE-11, and KRP-6.

ID	MW	QPlogPo/w	QPPCaco	QPPMDCK	PSA
TE-91	250.296	3.175	1157.299	579.332	49.51
TE-11	235.285	3.261	3148.242	1708.845	36.284
KRP-6	266.296	3.378	3673.745	2019.138	43.1

Abbreviations: MW: molecular weight, QPlogPo/w: predicted octanol/water partition coefficient (recommended values −2.0–6.5), QPPCaco: predicted apparent Caco-2 cell permeability in nm/s (recommended values <25 poor, >500 great), QPPMDCK: predicted apparent MDCK cell permeability in nm/s (<25 poor), PSA: polar surface area (van der Waals surface area for nitrogens, oxygens, and attached hydrogens).

## Data Availability

The original data presented in the study are openly available in The National Center for Biotechnology Information (NCBI) at https://www.ncbi.nlm.nih.gov (accessed on 9 October 2024).

## Supplementary Materials

The following supporting information can be downloaded at: https://www.mdpi.com/article/10.3390/antiox15050640/s1, Figure S1: Full-length western blot images; Figure S2: TE-91 inhibited ROS, nitrite, and IL-6 production in peritoneal macrophages; Figure S3: TE-91 inhibited ROS, nitrite, and IL-6 production in bone marrow-derived macrophages.
