# Supplementary material for: Selective MIF Enolase Inhibitor TE-91 Regulates M1 Polarization and Associated Metabolic Reprogramming
Source: Antioxidants (Basel). 2026 May 18;15(5):640. doi: 10.3390/antiox15050640 (PMC13203694; doi:10.3390/antiox15050640)
Supplement: Supplementary file 1 [file antioxidants-15-00640-s001.zip › antioxidants-4297074-supplementary.pdf]

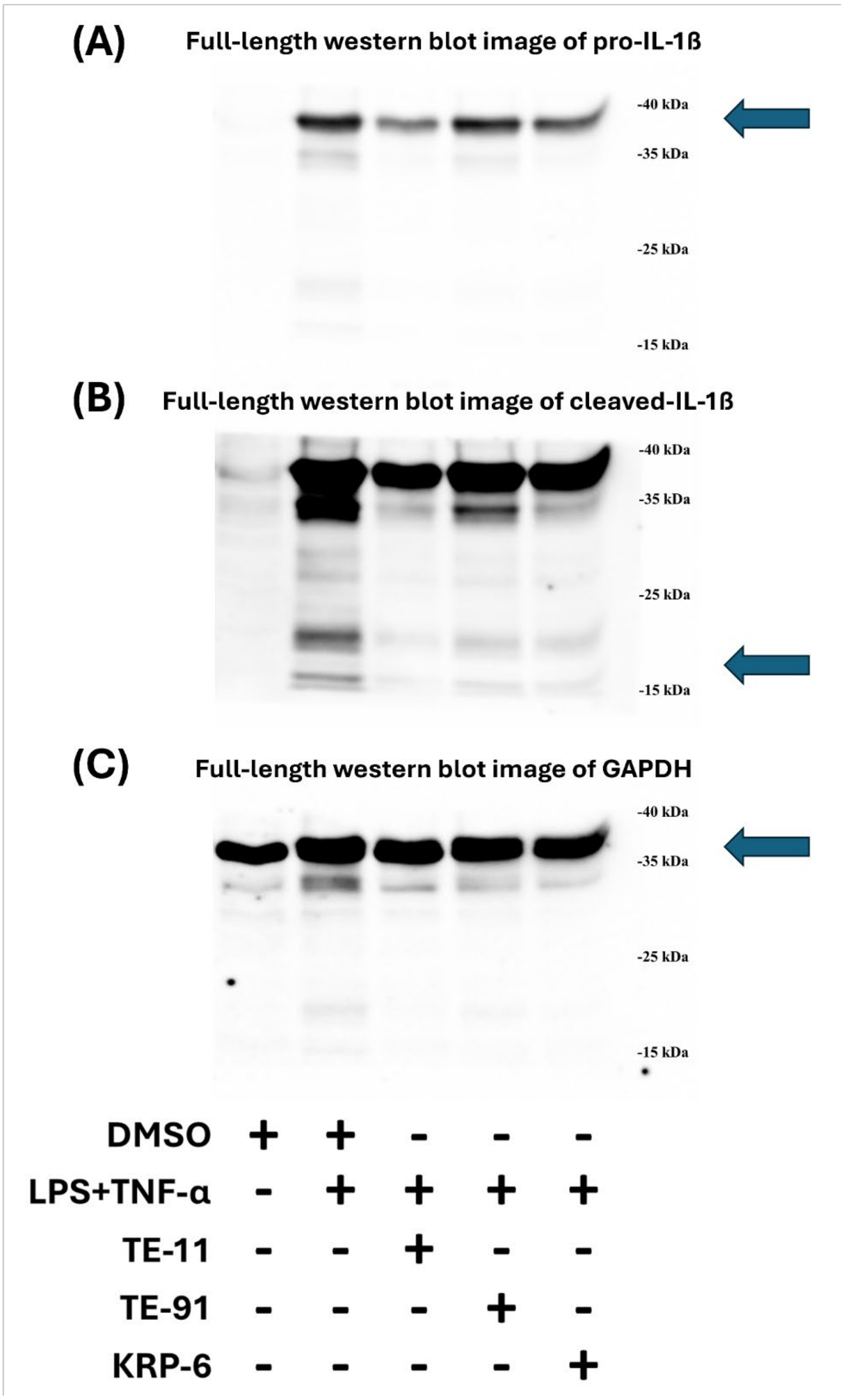

Supplementary Figure S1. Full-length western blot images. (**A**) pro-IL-1β, (**B**) cleaved-IL-1β, (**C**) stripped and reprobred membrane for GAPDH. Abbreviations: IL-1ß: interleukin-1ß, GAPDH: glyceraldehyde-3-phosphate, DMSO: dimethyl-sulfoxide, LPS: lipopolysaccharide, TNF-α: tumor necrosis factor-α.

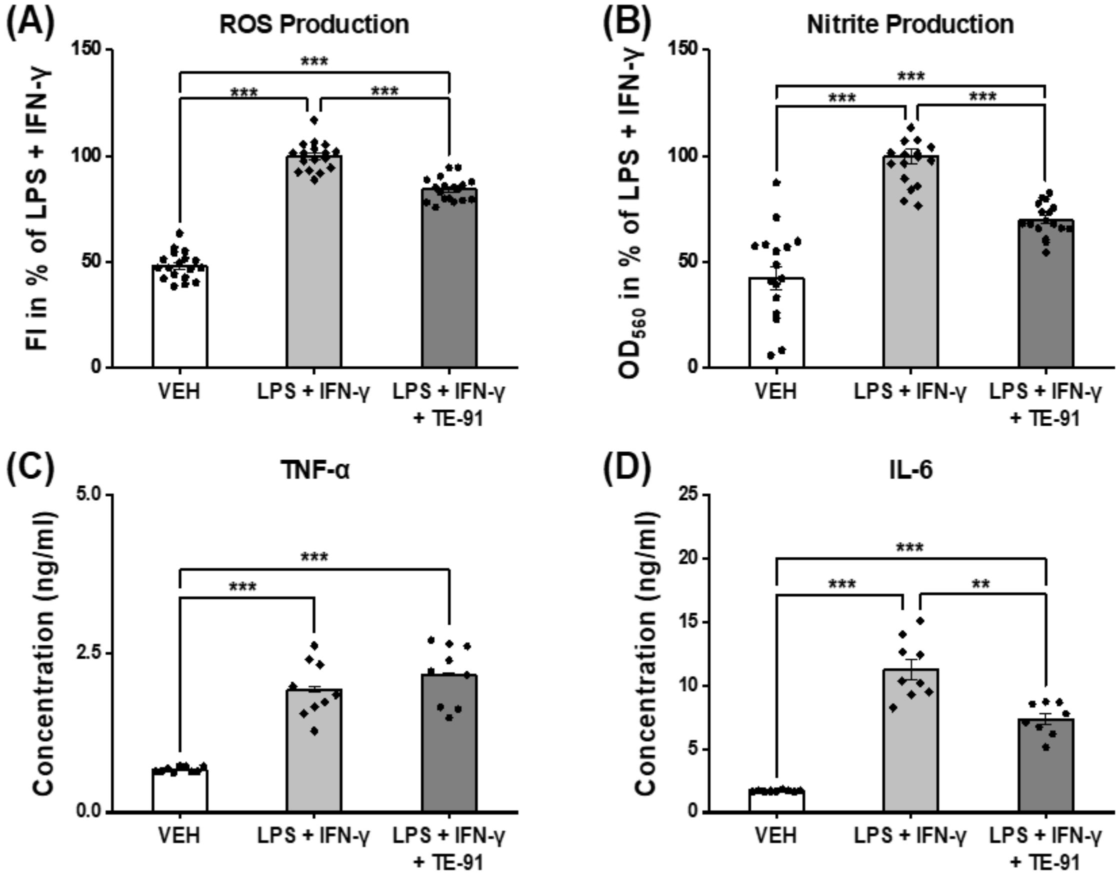

Supplementary Figure S2. TE-91 inhibited ROS, nitrite, and IL-6 production in peritoneal macrophages: Peritoneal macrophage (PM) cells were induced with 0.1 μg/ml LPS and 0.01 μg/ml IFN-γ for 24 hours after a 30 min pre-treatment with 20 μM TE-91. (**A**) ROS production was detected by adding 2 μM DHR123 fluorescent dye (490 nm excitation/ 510-570 nm emission). (**B**) Nitrite production was determined by mixing equal amounts of supernatant and Griess-Ilosvay reagent, and the optical density was measured at 560 nm. (**C**, **D**) TNF-*α* and IL-6 cytokine concentrations were measured from the supernatant via ELISA kits. Data are presented as (**A**, **B**) mean ± SEM in percent of LPS+IFN-γ treated groups, combined data of n=18 (3 independent experiments with 6 biological replicates), and (**C**, **D**) mean ± SEM, combined data of n=9 (3 independent experiments with 3 biological replicates). Statistical analyses were performed via (**A**, **B**) one-way ANOVA, and (**C**, **D**) Welch's ANOVA. ** $p<0.001$, *** $p<0.001$. Effect sizes: (**A**): $\eta 2=0.925$, (**B**): $\eta 2=0.674$, (**C**): $\eta 2=0.776$, (**D**): $\eta 2=0.882$. Abbreviations: VEH: vehicle, LPS: lipopolysaccharide, IFN-γ: interferon-γ, ROS: reactive oxygen species, TNF-*α*: tumor necrosis factor-*α*, IL-6: interleukin-6, DHR123: dihydrorhodamine 123, FI: fluorescent intensity, OD: optical density.

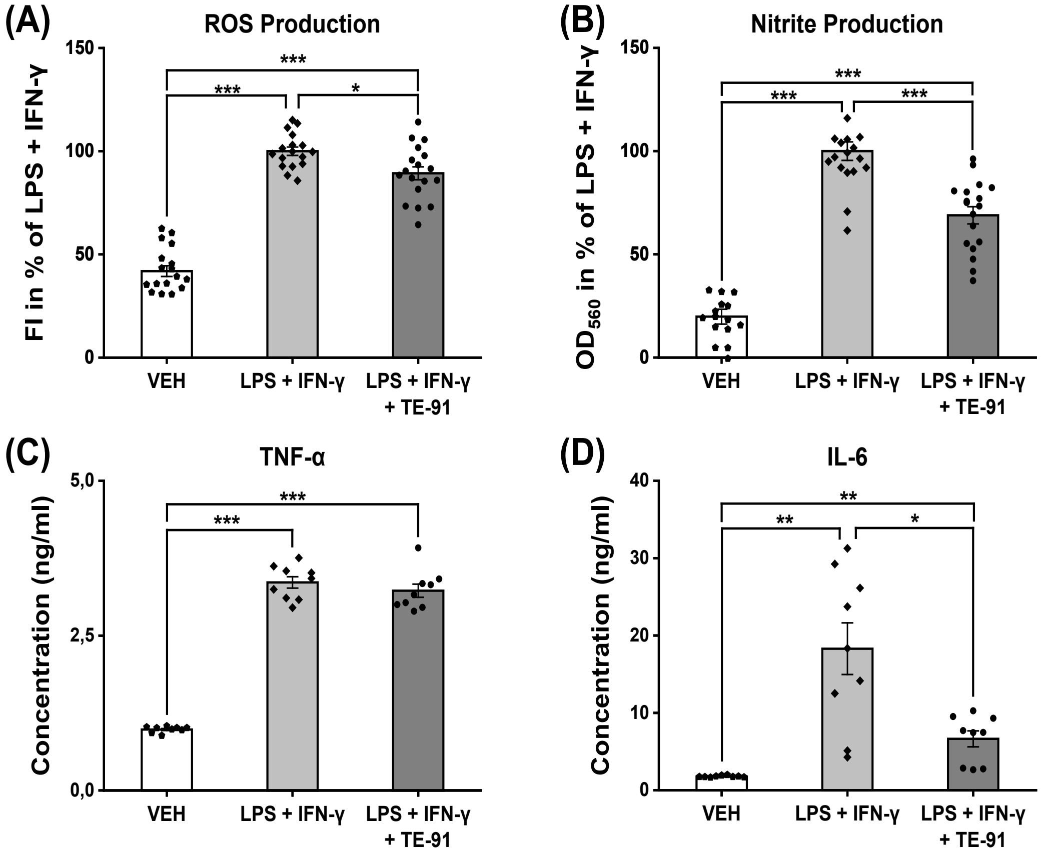

Supplementary Figure S3. TE-91 inhibited ROS, nitrite, and IL-6 production in bone marrow-derived macrophages: Bone marrow-derived macrophages (BMDMs) were pre-treated with 20 μM TE-91 for 30 min before 0.1 μg/ml LPS and 0.01 μg/ml IFN-γ treatment for another 24 hours. (**A**) ROS production was detected by adding 2 μM DHR123 fluorescent dye (490 nm excitation/ 510-570 nm emission). (**B**) Nitrite production was determined by mixing equal amounts of Griess-Ilosvay reagent and supernatant, and the optical density was measured at 560 nm. (**C**, **D**) TNF-α and IL-6 concentrations were measured from supernatant via ELISA. Data are presented as (**A**, **B**) mean ± SEM in percent of LPS+IFN-γ treated groups, combined data of n=18 (3 independent experiments with 6 biological replicates), and (**C**, **D**) mean ± SEM, combined data of n=9 (3 independent experiments with 3 biological replicates). Statistical analyses were performed via (**A**, **B**) one-way ANOVA, and (**C**, **D**) Welch's ANOVA. * $p<0.05$, ** $p<0.001$, *** $p<0.001$. Effect sizes: (**A**): $\eta 2=0.847$, (**B**): $\eta 2=0.793$, (**C**): $\eta 2=0.957$, (**D**): $\eta 2=0.597$. Abbreviations: VEH: vehicle, LPS: lipopolysaccharide, IFN-γ: interferon-γ, ROS: reactive oxygen species, TNF-α: tumor necrosis factor-α, IL-6: interleukin-6, DHR123: dihydrorhodamine 123, FI: fluorescent intensity, OD: optical density.
